# Ablative Management of Persistent Atrial Fibrillation (PeAF) with Posterior Wall Isolation (PWI): Where Do We Stand?

**DOI:** 10.3390/jcdd10070273

**Published:** 2023-06-27

**Authors:** Omar Baqal, Hicham Z. El Masry

**Affiliations:** 1 Department of Internal Medicine, Mayo Clinic, Phoenix, AZ 85054, USA; baqal.omar@mayo.edu; 2 Department of Cardiovascular Diseases, Mayo Clinic, Phoenix, 5777 E Mayo Blvd, Phoenix, AZ 85054, USA

**Keywords:** persistent atrial fibrillation, ablation therapy, posterior wall isolation, advanced ablation technologies

## Abstract

Atrial fibrillation is a diverse clinical entity, with persistent atrial fibrillation (PeAF) being particularly challenging to manage. Through this paper, we discuss notable developments in our understanding of ablative strategies for managing PeAF, with a special focus on posterior wall isolation (PWI).

## 1. Introduction

With advancements in ablative therapies and growing scientific evidence supporting the early implementation of ablation in the management of atrial fibrillation (AF), the debate over rate versus rhythm control has tilted toward the latter [1,2]. Although pulmonary vein isolation (PVI) has been the cornerstone of ablative therapy in AF, the recurrence rate of atrial arrhythmia after ablation continues to be a clinical challenge. Additional ablative targets have emerged and come under investigation, including the posterior left atrial wall, which is thought to play an important role in the initiation and maintenance of AF. The posterior left atrial wall shares its embryological origin with the pulmonary veins, both emerging from the mediastinal myocardium. The posterior wall also has shorter action potential durations and slower phase 0 upstroke velocity, with a higher propensity for fibrosis-related conduction delay [3]. The 2017 HRS/EHRA/ECAS/APHRS/SOLAECE expert consensus statement suggests posterior wall isolation (PWI) might be considered in initial or repeat ablation in persistent AF (PeAF) or long-standing PeAF (Class IIb) [4].

## 2. Posterior Wall Isolation in Persistent Atrial Fibrillation

**Figure 1:**
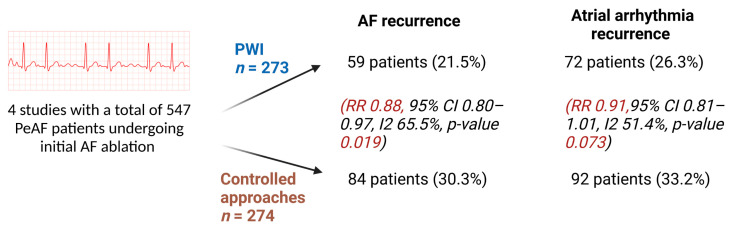
Visual illustration of outcomes in PeAF patients described in a meta-analysis by Kanitsoraphan et al. [11] (created with BioRender).

Although several cohort studies and small randomized trials have previously supported the inclusion of posterior wall isolation (PWI) and PVI in patients with persistent AF, data based on large-scale randomized trials are limited [5,6,7,8,9,10]. Kanitsoraphan et al. published a meta-analysis involving eight RCTs and 1024 patients and found that PWI did not significantly decrease overall atrial arrhythmia recurrences (RR 0.96, 95% CI 0.88–1.05, *I*^2^ = 31.6%, *p*-value 0.393): although PWI decreased AF recurrence compared to controlled approaches based on a pooled analysis (RR 0.88, 95% CI: 0.81–0.96, *I*^2^ = 48.2%, *p*-value 0.004), this benefit appears to be offset by increased incidence of atrial flutter after PWI [11]. In this meta-analysis, a decrease in AF recurrence was noted in the studies that included only persistent AF based on subgroup analysis (RR = 0.89, 95% CI: 0.80–0.98, *I*^2^ = 65.2%, *p*-value 0.014) [11] (Figure 1). No significant differences were noted in complications between PWI and control approaches, including risk of vascular access (RR 1.02, 95% CI 0.99–1.04, *I*^2^ 0.0%, *p*-value 0.222), pericardial effusion (RR 0.98, 95% CI 0.96–1.01, *I*^2^ 0.0%, *p*-value 0.147), pericarditis (RR 1.02, 95% CI 0.99–1.04, *I*^2^ 1.8%, *p*-value 0.240), and stroke or TIA (RR 1.00, 95% CI 0.97–1.03, *I*^2^ 0.0%, *p*-value 0.950) [11]. A 2022 stratified pooled analysis including 26 studies with 3287 patients with AF found that adjunctive PWI significantly lowered the recurrence of all atrial arrhythmias (risk ratio: 0.74; *p* < 0.001) and AF (risk ratio: 0.67; *p* ¼ 0.01) in PeAF, with patients with older age, large left atrial diameter and PeAF benefiting most from adjunctive PWI [12].

**Figure 2:**
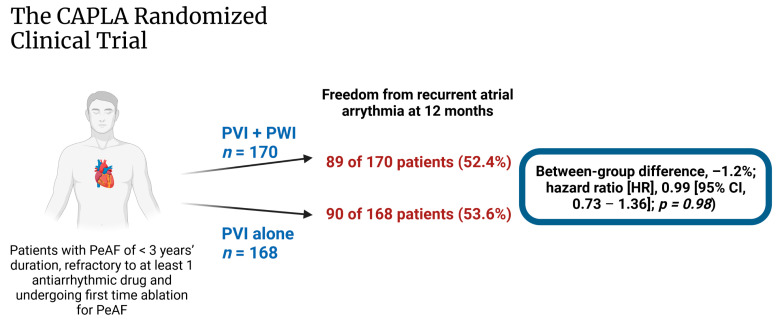
Illustrated summary of the CAPLA trial [13] (created with BioRender).

CAPLA, a recent randomized controlled trial involving 338 patients, compared PVI with PWI vs. PVI alone in patients with persistent AF of less than three years duration in whom one or more antiarrhythmic drugs had failed [13] (Figure 2). The primary endpoint, which consisted of freedom from any documented atrial arrhythmia of more than 30 s without antiarrhythmic medication at 12 months, was achieved in 89 patients (52.4%) assigned to PVI with PWI compared with 90 (53.6%) assigned to PVI alone (between-group difference, –1.2%; hazard ratio [HR], 0.99 [95% CI, 0.73–1.36]; *p* = 0.98). In essence, the empirical addition of PWI to PVI alone did not significantly improve freedom from atrial arrhythmia at 12 months in patients undergoing first-time catheter ablation for persistent AF.

The CAPLA trial is an important yet disappointing study for PWI proponents. The study findings are to be viewed in light of several limitations. Post-ablation rhythm monitoring was not homogenous. Likewise, pre-ablation AF burden was not reported, as rhythm monitoring prior to study enrollment was not standardized across all participants. Operators could not be blinded to randomization given the nature of the study, although they were also not involved in study endpoint interpretation.

## 3. The Role of Surgical Ablation

In an effort to improve procedural outcomes, operators may be tempted to attempt more extensive ablation, although this can be associated with increased procedural risk and complications, as well as longer procedural times [14]. Surgical ablation has been considered as a treatment option for patients requiring extensive ablation or patients who have failed PVI, as seen in patients with PeAF who experience considerable morbidity with catheter ablation and in whom long-term freedom from AF is less common than patients with paroxysmal AF [15,16,17]. Since its advent, the Cox maze procedure has evolved, incorporating new ablation technologies and minimally invasive approaches [18,19]. A meta-analysis involving eight studies and 744 patients comparing surgical and catheter ablation for atrial fibrillation noted that surgical ablation was associated with significantly lower arrhythmia recurrence (pooled HR 0.40 [0.35, 0.45], *p* < 0.001; low heterogeneity *I*^2^ 22%, *p* = 0.25), although the higher risk of major adverse events (death, myocardial infarction, coronary artery bypass surgery, and stroke) (pooled OR of 4.11 [2.26, 7.50] *p* < 0.001; low heterogeneity *I²* 0%, *p* = 0.51), longer procedural time (pooled mean difference of 41.17 min [4.14, 78.20], *p* = 0.03; high heterogeneity *I*^2^ 96% *p* < 0.001), and longer hospital stay (mean difference of 3.97 days [2.00, 5.95] *p* < 0.001; high heterogeneity *I*^2^ 88%, *p* < 0.001) [20].

Another recent meta-analysis by Rattanawong et al. compared video-assisted thoracoscopic and catheter radiofrequency pulmonary vein ablation included six studies from 2013 to 2020 involving 511 AF patients and found that catheter ablation was associated with increased atrial arrhythmia recurrence (pooled relative risk = 1.85, 95% confidence interval: 1.44–2.39, *p* < 0.001, *I*^2^ = 0.0%) but less total major adverse events (pooled relative risk = 0.29, 95% confidence interval: 0.16–0.53, *p* < 0.001, *I*^2^ = 0.0%) [21]. Catheter ablation was also associated with increased AF recurrence in refractory paroxysmal AF when compared to surgical ablation in the sub-group analysis (pooled relative risk = 2.47, 95% confidence interval: 1.31–4.65, *p* = 0.005, *I*^2^ = 0.0%) but not in persistent AF (relative risk = 1.09, 95% confidence interval: 0.60–2.0, *p* = 0.773) [21].

Based on six studies on minimally invasive surgical ablation and 56 on catheter ablation involving 7624 patients with PeAF, a systematic review and meta-analysis by Berger et al. noted that freedom from AF at 12 months was 69% (95% CI 64–74%) after surgical ablation and 51% (95% CI 46–56%) after catheter ablation, although adverse events occurred more frequently with surgical ablation than catheter ablation [22]. Adverse events after catheter ablation were mortality during the study course (1.1%), procedure-related death (0.1%), pacemaker implantations (0.9%), any bleeding (1.7%), pericarditis (1.4%) and thrombo–embolic events (0.7%) [20]. Adverse events after surgical ablation were mortality (1.1%), procedure-related death (0%), pacemaker implantation (2.7%), combined major and minor bleeding (7.7%), pneumothorax (6.1%), thrombo–embolic events (1.4%), and eight patients (1.6%) were converted to sternotomy [21]. It is important to note that all meta-analyses demonstrated considerable heterogeneity (*I*^2^ > 40%), with different catheter ablation approaches used across studies, while the minimally invasive surgical ablation strategies remained relatively uniform [22].

Although surgical ablation may confer longer arrhythmia-free times in certain patient populations, the higher risk of adverse events and longer postprocedure recovery must be considered when deciding to pursue surgical ablation over catheter ablation. Additionally, the lack of superiority of surgical ablation over catheter ablation as well as the demonstration of noninferiority of catheter ablation in notable studies such as the CASA-AF randomized controlled trial, must be acknowledged [23,24].

## 4. The Advent of Hybrid Endocardial/Epicardial Ablation

Although transvenous catheter ablation is a well-established management approach with good outcomes, especially in paroxysmal AF, its effectiveness in PeAF is limited, with rather disappointing results, independent of the ablation strategy used [14,25]. Although mechanisms of PeAF are complex and poorly understood, the advancements in our understanding of the phenomena of endocardial–epicardial dissociation (EED), discordant wavefront (DWF) patterns, and an epicardial breakthrough could help explain why endocardial ablation alone is often not enough in PeAF [26,27]. In an effort to achieve “best of both worlds”, the hybrid ablation (HA), combining a thoracoscopic epicardial and transvenous endocardial approach, was developed as a collaborative approach to the treatment of atrial fibrillation between cardiac surgeons and electrophysiologists. 

The CONVERGE trial (Convergence of Epicardial and Endocardial Ablation for the Treatment of Symptomatic Persistent AF), a prospective, multicenter, randomized controlled trial, compared the effectiveness and safety of the Hybrid Convergent procedure to endocardial catheter ablation in patients with symptomatic persistent and long-standing persistent AF [28]. One-hundred and fifty-three patients were randomized 2:1 to Hybrid Convergent versus CA, with 149 patients evaluable at 12 months. The Hybrid Convergent approach was found to have superior effectiveness (freedom from AF/atrial flutter/atrial tachycardia absent new/increased dosage of previously failed/intolerant class I/III antiarrhythmic drugs through 12 months) compared to CA (67.7% (67/99) vs. 50.0% (25/50) (*p* = 0.036) on/off previously failed antiarrhythmic drugs), with an acceptable safety profile [28]. A notable limitation of the study is the absence of empirical endocardial posterior wall ablation in the catheter ablation group, while the Hybrid Convergent arm received epicardial posterior wall silencing. 

A recent prospective, superiority, unblinded, randomized controlled trial comparing the effectiveness and safety of HA with CA in 41 ablation-naive patients with (long-standing)-PeAF highlighted higher freedom from atrial tachyarrhythmias off antiarrhythmic drugs in the HA group compared with the CA group (89% vs. 41%, *p* = 0.002), with comparable serious adverse event rates (21% vs. 14%, *p* = 0.685) and quality of life (QoL) scores between the two groups (*p* = 0.491) [29]. A meta-analysis by Shrestha et al. investigating the effectiveness of Hybrid Convergent ablation in patients mostly with drug-refractory PeAF and long-standing PeAF noted that freedom from atrial arrhythmias was 69% (95% confidence interval [CI]: 61–78%, *n* =523) and 50% (95% CI: 42–58%, *n* = 343) off antiarrhythmic drugs [30]. The thirty-day major adverse event rate was 6% (95% CI: 3–8%, *n* = 551), with individual major adverse event rates comparable to estimates from AF-related ablation [4,30]. Considerable heterogeneity was noted across studies, including in the extent of endocardial ablation, rhythm monitoring, and history of prior ablation [30].

## 5. Our Opinion

When it comes to ablative management of AF, especially PeAF, we currently do not have a “one size fits all” solution. Current evidence on the benefit of adjunctive PWI in the management of PeAF remains contentious, at best. With additional ablation targets and a longer procedure duration, the potential of increased complications should be taken into consideration, namely the dreadful atrio-esophageal fistula and subclinical cerebral embolism. Both of these complications are difficult to adjudicate in most published clinical studies. The use of the esophageal cooling probe and esophageal deviation are among the measures implemented to reduce the risk of atrio-esophageal fistula, although data remain scarce on the effectiveness of such measures given the rarity of the complication. Per current guidelines, posterior wall isolation (PWI) may be considered in initial or repeat ablation in persistent AF (PeAF) or long-standing PeAF (Class IIb), whereas stand-alone surgical ablation is reasonable for patients with PeAF and long-standing PeAF, who have failed one or more attempts at catheter ablation, and after review of the relative safety and efficacy of catheter ablation versus a stand-alone surgical approach for those who are intolerant or refractory to antiarrhythmic therapy and prefer a surgical approach (Class IIa). Per the expert statement, it is reasonable to apply the same indications for stand-alone surgical ablation to patients being considered for hybrid surgical ablation (Class IIb) [4]. It remains unclear which PeAF patient subgroups would benefit most from adjunctive PWI and which ablation-naïve PeAF patients would benefit most from direct escalation to surgical/hybrid ablation. 

Currently, we recommend against empiric PWI, given the paucity of data. Our approach has focused on attempting to risk stratify the PeAF phenotype by evaluating a multitude of risk factors of arrhythmia recurrence, including the duration of AF, atrial volume and function, and the clinical comorbidities prior to ablation. We also invest significant effort to restore sinus rhythm by an escalation of antiarrhythmic therapy prior to the ablation and then intraprocedurally assessing the atrial substrate by voltage mapping: only patients with significant low voltage in the posterior wall PWI are considered. This approach has been particularly influenced by promising studies employing a substrate-guided approach to PeAF ablation [31]. The left atrial (LA) posterior wall has been shown to have the highest proportion of non-PV triggers, and those triggers remain important targets of ablative management, falling under the domain of targeted focal ablation rather than PWI [32]. It also houses the septopulmonary bundle (SPB), which is considered a site of conduction slowing and wavefront collision and can pose challenges to PWI [33,34]. At the thickest region of the SPB, the bidirectional block can be difficult to achieve, with SBP thickness decreasing considerably between the superior pulmonary veins and further between the inferior pulmonary veins. If the electrical connection persists after the completion of the roof and inferior lines, both lines are mapped and ablated, followed by a direct approach to the roof line with a steerable sheath. Then, pacing from the ablation catheter positioned immediately anterior and posterior to the roof line is performed with a circular mapping catheter [35]. A study involving 100 patients undergoing atrial fibrillation ablation (90% de novo ablation for PeAF, 10% repeat ablation for recurrent paroxysmal AF) noted that residual gaps were more frequent in roof lines than floor lines (33% vs. 15%; *p* = 0.049), highlighting the importance of careful electrogram analysis and role of floor line as an effective alternative strategy [36].

Further studies are needed to evaluate the utility of adjunctive PWI as well as other ablation approaches, including surgical ablation, in the management of PeAF. Future studies could explore the impact of operator characteristics and experience, and institutional characteristics on the choice of ablation approach and clinical outcomes. The scientific inquiry into the complex three-dimensional electrophysiological mechanisms behind PeAF must continue. As we grow in our understanding of the differences in outcomes with various ablation approaches, it remains important to personalize the decision in the context of important factors for patient clinical characteristics such as age, comorbidities, AF duration, risk of adverse events, and cost-effectiveness.

## Data Availability

No new data generated.

## References

[1] Wazni O.M., Dandamudi G., Sood N., Hoyt R., Tyler J., Durrani S., Niebauer M., Makati K., Halperin B., Gauri A. (2021). Cryoballoon ablation as initial therapy for atrial fibrillation. N. Engl. J. Med..

[2] Kirchhof P., Camm A.J., Goette A., Brandes A., Eckardt L., Elvan A., Fetsch T., van Gelder I.C., Haase D., Haegeli L.M. (2020). Early rhythm-control therapy in patients with atrial fibrillation. N. Engl. J. Med..

[3] Ehrlich J.R., Cha T.J., Zhang L., Chartier D., Melnyk P., Hohnloser S.H., Nattel S. (2003). Cellular electrophysiology of canine pulmonary vein cardiomyocytes: Action potential and ionic current properties. J. Physiol..

[4] Calkins H., Hindricks G., Cappato R., Kim Y.H., Saad E.B., Aguinaga L., Akar J.G., Badhwar V., Brugada J., Camm J. (2017). 2017 HRS/EHRA/ECAS/APHRS/SOLAECE expert consensus statement on catheter and surgical ablation of atrial fibrillation. Heart Rhythm.

[5] Salih M., Darrat Y., Ibrahim A.M., Al-Akchar M., Bhattarai M., Do C.K., Ayan M., Labedi M., Elayi C.S. (2020). Clinical outcomes of adjunctive posterior wall isolation in persistent atrial fibrillation: A meta-analysis. J. Cardiovasc. Electrophysiol..

[6] Ahn J., Shin D.G., Han S.J., Lim H.E. (2022). Does isolation of the left atrial posterior wall using cryoballoon ablation improve clinical outcomes in patients with persistent atrial fibrillation? A prospective randomized controlled trial. Europace.

[7] Cutler M.J., Johnson J., Abozguia K., Rowan S., Lewis W., Costantini O., Natale A., Ziv O. (2016). Impact of voltage mapping to guide whether to perform ablation of the posterior wall in patients with persistent atrial fibrillation. J. Cardiovasc. Electrophysiol..

[8] Bai R., Di Biase L., Mohanty P., Trivedi C., Russo A.D., Themistoclakis S., Casella M., Santarelli P., Fassini G., Santangeli P. (2016). Proven isolation of the pulmonary vein antrum with or without left atrial posterior wall isolation in patients with persistent atrial fibrillation. Heart Rhythm.

[9] Kim J.-S., Shin S.Y., Na J.O., Choi C.U., Kim S.H., Kim E.J., Rha S.-W., Park C.G., Seo H.S., Oh D.J. (2015). Does isolation of the left atrial posterior wall improve clinical outcomes after radiofrequency catheter ablation for persistent atrial fibrillation? A prospective randomized clinical trial. Int. J. Cardiol..

[10] O’Neill L., Hensey M., Nolan W., Keane D. (2015). Clinical outcome when left atrial posterior wall box isolation is included as a catheter ablation strategy in patients with persistent atrial fibrillation. J. Interv. Card. Electrophysiol..

[11] Kanitsoraphan C., Rattanawong P., Techorueangwiwat C., Kewcharoen J., Mekritthikrai R., Prasitlumkum N., Shah P., El Masry H. (2022). The efficacy of posterior wall isolation in atrial fibrillation ablation: A systematic review and meta-analysis of randomized controlled trials. J. Arrhythm..

[12] Jiang X., Liao J., Ling Z., Meyer C., Sommer P., Futyma P., Martinek M., Schratter A., Acou W.-J., Wang J. (2022). Adjunctive Left Atrial Posterior Wall Isolation in Treating Atrial Fibrillation: Insight from a Large Secondary Analysis. JACC Clin. Electrophysiol..

[13] Kistler P.M., Chieng D., Sugumar H., Ling L.-H., Segan L., Azzopardi S., Al-Kaisey A., Parameswaran R., Anderson R.D., Hawson J. (2023). Effect of catheter ablation using pulmonary vein isolation with vs without posterior left atrial wall isolation on atrial arrhythmia recurrence in patients with persistent atrial fibrillation: The CAPLA randomized clinical trial. JAMA.

[14] Verma A., Jiang C.-Y., Betts T.R., Chen J., Deisenhofer I., Mantovan R., Macle L., Morillo C.A., Haverkamp W., Weerasooriya R. (2015). Approaches to catheter ablation for persistent atrial fibrillation. N. Engl. J. Med..

[15] Patsopoulos N.A., Evangelou E., Ioannidis J.P. (2008). Sensitivity of between-study heterogeneity in meta-analysis: Proposed metrics and empirical evaluation. Int. J. Epidemiol..

[16] Scherr D., Khairy P., Miyazaki S., Aurillac-Lavignolle V., Pascale P., Wilton S.B., Ramoul K., Komatsu Y., Roten L., Jadidi A. (2015). Five-year outcome of catheter ablation of persistent atrial fibrillation using termination of atrial fibrillation as a procedural endpoint. Circ. Arrhythm. Electrophysiol..

[17] Tilz R.R., Rillig A., Thum A.-M., Arya A., Wohlmuth P., Metzner A., Mathew S., Yoshiga Y., Wissner E., Kuck K.-H. (2012). Catheter ablation of long-standing persistent atrial fibrillation: 5-year outcomes of the Hamburg Sequential Ablation Strategy. J. Am. Coll. Cardiol..

[18] Damiano R.J., Bailey M. (2007). The Cox-Maze IV procedure for lone atrial fibrillation. Multimed. Man. Cardiothorac. Surg..

[19] Cox J.L., Ad N. (2000). New surgical and catheter-based modifications of the Maze procedure. Semin. Thorac. Cardiovasc. Surg..

[20] Yonas E., Pranata R., Siswanto B.B., Abdulgani H.B. (2020). Comparison between surgical and catheter based ablation in atrial fibrillation, should surgical based ablation be implemented as first line? A meta-analysis of studies. Indian Pacing Electrophysiol. J..

[21] Rattanawong P., Kanitsoraphan C., Kewcharoen J., Sriramoju A., Shanbhag A., Ko N.L.K., Barry T., Vutthikraivit W., Shen W. (2022). Surgical versus catheter ablation in atrial fibrillation: A systematic review and meta-analysis of randomized controlled trials. J. Cardiovasc. Electrophysiol..

[22] Berger W.R., Meulendijks E.R., Limpens J., Berg N.W.V.D., Neefs J., Driessen A.H., Krul S.P., van Boven W.J.P., de Groot J.R. (2019). Persistent atrial fibrillation: A systematic review and meta-analysis of invasive strategies. Int. J. Cardiol..

[23] Haldar S., Khan H.R., Boyalla V., Kralj-Hans I., Jones S., Lord J., Onyimadu O., Satishkumar A., Bahrami T., De Souza A. (2020). Catheter ablation vs. thoracoscopic surgical ablation in long-standing persistent atrial fibrillation: CASA-AF randomized controlled trial. Eur. Heart J..

[24] Adiyaman A., Buist T.J., Beukema R.J., Smit J.J.J., Delnoy P.P.H., Hemels M.E., Sie H.T., Misier A.R.R., Elvan A. (2018). Randomized Controlled Trial of Surgical Versus Catheter Ablation for Paroxysmal and Early Persistent Atrial Fibrillation. Circ. Arrhythm. Electrophysiol..

[25] Nault I., Miyazaki S., Forclaz A., Wright M., Jadidi A., Jaïs P., Hocini M., Haïssaguerre M. (2010). Drugs vs. ablation for the treatment of atrial fibrillation: The evidence supporting catheter ablation. Eur. Heart J..

[26] Aronis K.N., Trayanova N.A. (2020). Endocardial-Epicardial Dissociation in Persistent Atrial Fibrillation: Driver or Bystander Activation Pattern?. Circ. Arrhythm. Electrophysiol..

[27] Parameswaran R., Kalman J.M., Royse A., Goldblatt J., Larobina M., Watts T., Walters T.E., Nalliah C.J., Wong G., Al-Kaisey A. (2020). Endocardial-Epicardial Phase Mapping of Prolonged Persistent Atrial Fibrillation Recordings: High Prevalence of Dissociated Activation Patterns. Circ. Arrhythm. Electrophysiol..

[28] Delurgio D.B., Crossen K.J., Gill J., Blauth C., Oza S.R., Magnano A.R., Mostovych M.A., Halkos M.E., Tschopp D.R., Kerendi F. (2020). Hybrid Convergent Procedure for the Treatment of Persistent and Long-Standing Persistent Atrial Fibrillation: Results of CONVERGE Clinical Trial. Circ. Arrhythm. Electrophysiol..

[29] Van der Heijden C.A., Weberndörfer V., Vroomen M., Luermans J.G., Chaldoupi S.-M., Bidar E., Vernooy K., Maessen J.G., Pison L., van Kuijk S.M. (2023). Hybrid Ablation Versus Repeated Catheter Ablation in Persistent Atrial Fibrillation: A Randomized Controlled Trial. JACC Clin. Electrophysiol.

[30] Shrestha S., Plasseraud K.M., Makati K., Sood N., Killu A.M., Contractor T., Ahsan S., De Lurgio D.B., Shults C.C., Eldadah Z.A. (2022). Hybrid Convergent ablation for atrial fibrillation: A systematic review and meta-analysis. Heart Rhythm O2.

[31] Huo Y., Gaspar T., Schönbauer R., Wójcik M., Fiedler L., Roithinger F.X., Martinek M., Pürerfellner H., Kirstein B., Richter U. (2022). Low-voltage myocardium-guided ablation trial of persistent atrial fibrillation. NEJM Evid..

[32] Lin W.-S., Tai C.-T., Hsieh M.-H., Tsai C.-F., Lin Y.-K., Tsao H.-M., Huang J.-L., Yu W.-C., Yang S.-P., Ding Y.-A. (2003). Catheter ablation of paroxysmal atrial fibrillation initiated by non-pulmonary vein ectopy. Circulation.

[33] Markides V., Schilling R.J., Ho S.Y., Chow A.W., Davies D.W., Peters N.S. (2003). Characterization of left atrial activation in the intact human heart. Circulation.

[34] Roberts-Thomson K.C., Stevenson I.H., Kistler P.M., Haqqani H.M., Goldblatt J.C., Sanders P., Kalman J.M. (2008). Anatomically determined functional conduction delay in the posterior left atrium relationship to structural heart disease. J. Am. Coll. Cardiol..

[35] Sugumar H., Thomas S.P., Prabhu S., Voskoboinik A., Kistler P.M. (2018). How to perform posterior wall isolation in catheter ablation for atrial fibrillation. J. Cardiovasc. Electrophysiol..

[36] Pambrun T., Duchateau J., Delgove A., Denis A., Constantin M., Ramirez F.D., Chauvel R., Tixier R., Welte N., André C. (2021). Epicardial course of the septopulmonary bundle: Anatomical considerations and clinical implications for roof line completion. Heart Rhythm.

